# Fractional-order stochastic delayed neural networks with impulses: mean square finite-time contractive synchronization

**DOI:** 10.1038/s41598-025-31768-7

**Published:** 2025-12-10

**Authors:** Gokul Palanisamy, Udhayakumar Kandasamy, Fathalla A. Rihan, Salem Ben Said

**Affiliations:** Department of Mathematical Sciences, College of Science, United Arab Emirates University, AL Ain, UAE

**Keywords:** Neural networks, Caputo-fractional derivative, Impulses, Time delays, Mean square synchronization, Contractive peoperty, Engineering, Mathematics and computing, Physics

## Abstract

This article presents a novel framework for mean square finite-time synchronization (MSFTSn) and mean square finite-time contractive synchronization (MSFTCSn) of fractional-order stochastic delayed neural networks (FOSDNNs) subject to hybrid control. The proposed hybrid control strategy is designed to guarantee synchronization of the error system within a finite time horizon. By combining continuous feedback with impulsive regulation, the hybrid mechanism effectively suppresses stochastic disturbances and compensates for time-delay effects, which significantly improves convergence rate and enhances contractive stability. The analytical approach integrates stochastic analysis with Lyapunov-based methods, the fractional Gronwall inequality, and an improved Razumikhin framework to establish novel synchronization criteria. In addition, a rigorous foundation is developed to address discontinuous neuron activation functions through set-valued map theory. Unlike integer-order models, the Caputo fractional derivative embeds past error trajectories, thereby capturing memory and hereditary properties of neural systems. This leads to a more realistic neural representation and reinforces the synchronization results. Theoretical findings demonstrate that hybrid control extends the range of stabilizing parameters beyond standard feedback schemes. Finally, numerical simulations are presented to validate the effectiveness and robustness of the proposed strategy, confirming its strong applicability in realistic neural network models.

## Introduction

Neural networks (NNs) have emerged as powerful tools in computational models due to their capability of approximation, adaptive learning, and non-linear mapping efficiency. They are extensively applied in control, optimization, and signal processing, where conventional methods face challenges arising from system complexity or incomplete modelling^1–4^. In recent years, integer-order stochastic NNs have been widely adopted in diverse engineering and scientific fields due to their strong modeling capability under uncertainty. They have been successfully utilized for climate and financial forecasting through stochastic process adaptation^5^, for enhancing computational efficiency in machine learning via stochastic computing architectures^6^, and for ensuring secure communication using synchronization-based control schemes^7^. These applications highlight the effectiveness of stochastic NNs in handling randomness, time delays, and uncertain dynamics in complex real-world systems. However, traditional NNs are inherently based on integer-order dynamics and thus may not adequately capture the memory and hereditary characteristics present in many physical and biological processes^8–10^. To tackle this, fractional calculus has been integrated into the neural network framework, which in turn gives rise to fractional-order NNs. By incorporating fractional derivatives into activation dynamics and learning rules, fractional-order NNs introduce non-local and memory-dependent behavior, which boosts convergence speed, stability, and generalization compared to classical NNs^11–14^. This extension not only enriches the representational power of neural architectures but also integrates seamlessly with fractional-order control systems, making fractional-order NNs a robust framework for intelligent modelling and control in complex, uncertain, and memory-driven environments. Evolving from this foundation, researchers have further extended the notion to fractional-order NNs with delay, in which time delays are introduced to more accurately represent systems that experience delayed feedback, control, communication lags, or transport phenomena^15–17^. These networks exhibit improved dynamic behavior, enhanced stability analysis, and superior modelling accuracy, thereby providing an effective paradigm for addressing real-world problems characterized by both fractional-order dynamics and time delays^18–20^.

Furthermore, building upon this, fractional-order stochastic NNs have been designed to deal with the memory-dependent characteristics of fractional calculus and the randomness of stochastic environments. Thus, by integrating these two aspects, fractional-order stochastic NNs achieve efficient modelling fidelity and adaptability, which makes them appropriate for uncertain systems that contain noise, perturbations, and randomness^21–23^. Recent studies have further demonstrated their wide applicability in real-world problems, including financial forecasting and macroeconomic analysis involving impulsive and stochastic effects^24,25^, fault-tolerant control of uncertain fractional-order neural systems with stochastic sensor faults^26^, and fractional stochastic partial differential equations for advanced scientific and engineering applications^27^. These studies emphasize the strong adaptability and effectiveness of fractional-order stochastic NNs in capturing hybrid dynamics influenced by randomness, delays, and impulsive behaviors. Besides, they outperform integer-order or solely deterministic NNs in terms of stability, faster convergence under changing input, and synchronization performance. Added to this, the significant branch of research concentrates on the role of discontinuous activation functions in fractional-order stochastic neural networks. Although such functions introduce analytical difficulties due to non-smooth state trajectories, they have been shown to significantly enhance synchronization and control performance, particularly in finite-time analysis^28^. These discontinuous mechanisms expand the applicability of fractional-order stochastic NNs to problems where there are sudden changes or switching characteristics that occur in the system dynamics^29–32^. Likewise, fractional-order stochastic NNs with delay extend this framework by incorporating the explicit time delays into the system. Time delays have major implications in effectively modelling communication lags in networked systems, transport delays in distributed processes, and feedback delays in biological and control systems. Also, fractional-order stochastic NNs with delay models enable the derivation of rigorous stability and synchronization criteria through Lyapunov-Krasovskii functionals, stochastic analysis, and fractional Gronwall inequalities, offering a mathematically sound framework for capturing stochastic, hereditary, and delay-dependent effects simultaneously.

As a result, they provide a complete and scalable approach to intelligent modelling and control in a variety of advanced applications, including robotics, power systems, biomedical engineering, and large-scale distributed networks^33–35^. In addition to stochastic disturbances and delays, many real processes are influenced by impulsive effects, which emerge as abrupt state changes caused by shocks, switching actions, or external perturbations. To capture such phenomena, the framework has been extended to fractional-order stochastic NNs with impulses, where impulsive differential operators are incorporated into the fractional stochastic setting. The incorporation of impulses improves the modelling capability of these networks, but it also poses considerable analytical hurdles, as the system trajectories are influenced simultaneously by fractional memory, stochastic disturbances, temporal delays, and instantaneous state jumps. To address these challenges, advanced tools like as piecewise Lyapunov functionals, impulsive integral inequalities, and stochastic analysis have been used to create sufficient criteria for stability, boundedness, and synchronization. As a result, fractional-order stochastic NNs with discontinuous activations, delays, and impulses offer a comprehensive paradigm for analyzing and controlling hybrid stochastic systems characterized by memory dependence, uncertainty, and abrupt dynamic variations, with applications ranging from power systems and communication networks to biomedical signal processing and robotic control^36^.

In modern neural and control system analysis, finite-time stability (FTS) ensures that trajectories reach equilibrium within a specified duration, offering faster convergence than asymptotic stability, which allows convergence only as time approaches infinity. This time-constrained behavior is particularly valuable for safety-critical and rapid-response applications where timely convergence is essential^37–42^. Moreover, the concept of finite-time contractive stability (FTCS) strengthens this framework by ensuring that the distance between any two trajectories diminishes within finite time, which is crucial for synchronization in large-scale or interconnected neural networks subject to modeling inaccuracies, uncertainties, and external disturbances^43–45^. When randomness and noise are present, mean-square stochastic finite-time stability extends this notion by analyzing convergence through second-order moments, ensuring that the expected squared deviation from equilibrium vanishes within a finite time frame. Furthermore, the integration of fractional calculus gives rise to mean-square stochastic fractional finite-time stability, where fractional-order derivatives effectively capture long-memory and non-local dynamics characteristic of many real-world systems^46–48^. Hence, the fusion of fractional operators with stochastic finite-time frameworks establishes a more flexible and realistic modeling paradigm, facilitating neural and control systems that achieve rapid convergence, efficient memory utilization, and enhanced robustness against stochastic perturbations.

Despite significant advancements in the stability analysis of stochastic and fractional-order NNs, numerous unresolved difficulties hinder their practical implementation in real-time and safety-critical contexts. The main challenges associated with fractional-order models compared with integer-order systems can be summarized as follows:

1. Fractional-order systems exhibit memory and hereditary characteristics, causing their current states to depend on historical trajectories, which complicates dynamic modeling and analytical formulation.
2. The presence of nonlocal fractional derivatives and stochastic effects increases the mathematical difficulty in establishing Lyapunov-based stability criteria and deriving feasible finite-time conditions.
3. Strong coupling among system states, along with sensitivity to the fractional derivative order, makes controller design and stability analysis more complex compared with integer-order NNs.

These inherent complexities significantly intensify the analytical and control design challenges in FOSDNNs. Although considerable progress has been achieved, most existing studies emphasize asymptotic or exponential stability, which guarantees convergence only over an infinite horizon and thus falls short for applications requiring rapid stabilization within a finite duration. While fractional calculus effectively captures long-memory effects and stochastic modeling enhances robustness against random perturbations, the combined influence of time delays and impulsive behaviors introduces additional dynamic complexity that remains insufficiently explored within finite-time constraints. This limitation highlights a critical research gap in developing a unified theoretical framework capable of simultaneously addressing MSFTSn and MSFTCSn for FOSDNNs under hybrid control. The motivation of this study, therefore, lies in bridging this gap by formulating a rigorous framework that establishes finite-time stability criteria, ensuring fast convergence, improved robustness, and stronger adaptability of hybrid-controlled NNs operating under stochastic disturbances, memory effects, delays, and impulsive influences. Based on this motivation, the key contributions of this study are summarized as follows:

1. A comprehensive hybrid control framework is proposed to overcome the analytical and design challenges of FOSDNNs by simultaneously achieving MSFTSn and MSFTCSn under the combined influence of stochastic disturbances, delays, impulses, and memory effects, thereby addressing the identified research gap in unified finite-time synchronization.
2. The developed approach employs Filippov set-valued mapping, free-weighting matrices, and advanced inequality techniques to manage both continuous and discontinuous activations, ensuring less conservative stability conditions and accurate finite-time convergence analysis.
3. The framework integrates fractional-order dynamics, hybrid control, and stochastic characteristics into a single formulation, effectively enhancing robustness, convergence speed, and adaptability in uncertain and time-delayed neural network environments, as demonstrated through detailed numerical simulations.

Furthermore, to facilitate subsequent analysis, the essential mathematical tools, notation, definitions, and lemmas used throughout the paper are presented in the following preliminaries section.

## Preliminaries

### System description

Consider the following fractional-order neural networks (FONNs) model:

1 $$\begin{aligned} \begin{aligned} {}^{C}_{0}D_{t}^{\beta } x(t)&= -A x(t) + B f\big (x(t)\big ) + C f\big (x(t - \tau (t))\big ), \\ x(t_0 + l)&= \delta (l). \end{aligned} \end{aligned}$$

Where $\beta \in (0,1)$ denotes the fractional order, $x(t) \in \mathbb {R}^n$ is the state vector of the FONNs (1); $A \in \mathbb {R}^{n \times n}$ represents a diagonal self-connection matrix; $B, C \in \mathbb {R}^{n \times n}$ correspond to the connection weight matrix, the delayed connection weight matrix, respectively. The function $f : \mathbb {R}^n \rightarrow \mathbb {R}^n$ denotes the neuron activation function, satisfying $f(0)=0$. The notation $\tau (t)$ represents the time varying delay, and it satisfies $0 \le \tau (t) \le t$. The initial function $\delta (l) \in PC_{\tau }$ is defined for $t_0-\tau \le l \le t_0$.

#### Assumption 1

The nonlinear function *f* in system (1) is assumed to satisfy

$$\begin{aligned} \mathscr {L}_1 \le \frac{f(\kappa _1)-f(\kappa _2)}{\kappa _1-\kappa _2} \le \mathscr {L}_2, \quad \forall \kappa _1, \kappa _2 \in \mathbb {R}, \ \kappa _1 \ne \kappa _2, \end{aligned}$$

where $f(0)=0$, and $\mathscr {L}_1, \mathscr {L}_2 \in \mathbb {R}^{n \times n}$ are diagonal matrices.

Let $\mathscr {K}$ and $\mathscr {M}$ be two separable Hilbert spaces, and $\mathscr {L}(\mathscr {K}, \mathscr {M})$ be the space of bounded linear operators from $\mathscr {K}$ into $\mathscr {M}$, $\mathscr {L}(\mathscr {K})=\mathscr {L}(\mathscr {K}, \mathscr {K}).$ $\Vert \cdot \Vert$ represents the norm in $\mathscr {K}, \mathscr {M}, \mathscr {L}(\mathscr {K}),$ and $\mathscr {L}(\mathscr {K}, \mathscr {M}).$ Let $(\Omega , \mathscr {F}, \{\mathscr {F}_t\}_{t \ge 0}, \mathscr {P})$ be a complete filtered probability space satisfying that $\mathscr {F}_0$ contains all $\mathscr {P}-$null sets of $\mathscr {F}.$ The noise-free system (1) is referred to as the drive system, and its corresponding response system can be expressed as follows:

2 $$\begin{aligned} \begin{aligned} {}_{0}^{C}D_{t}^{\beta } y(t)&= \left[ -A\,y(t) + B\,f(y(t)) + C\,f(y(t-\tau (t))) + D u(t) \right] + h(t, y(t)) \frac{{dB}^{H}(t)}{dt}, \\ y(t_0 + l)&= \epsilon (l). \end{aligned} \end{aligned}$$

Where, *D* represents the input weight matrix, $u(t) \in \mathbb {R}^n$ denotes the hybrid control input defined later, and $\epsilon (l) \in PC_{\tau }$. $h(\cdot , \cdot ) \in \mathscr {L}^2([0, \infty ) \times \mathscr {K}; \mathscr {L}(\mathscr {K}, \mathscr {M}))$ is continuous nonlinear mapping functions, with $h(\cdot , \cdot )$ is the noise intensity function. Let $B=\{B(t)\}_{t \ge 0}$ be a zero-mean Brownian motion stochastic perturbation defined on $(\Omega , \mathscr {F}, \{\mathscr {F}_t\}_{t \ge 0}, \mathscr {P})$, while fBm is a family of Gaussian processes indexed by Hurst parameter $B^H(t), H \in (0,1).$ According to^49^, a $\mathscr {K}-$value stochastic process $\{y(t)\}_{t \ge 0}$ is called a mild solution of the system (2), if the stochastic process $\{y(t)\}_{t \ge 0}$ is continuous and $\mathscr {F}_t$ adapted, the function $h(\cdot , \cdot ) \in \mathscr {L}^2([0, \infty ) \times \mathscr {K}; \mathscr {L}(\mathscr {K}, \mathscr {M}))$ and the $\beta$-order R-L fractional integral equation of (2) holds for every *t* with probability one.

In the following, the error system *e*(*t*) is derived from the master system (1) and the slave system (2), and its dynamics are expressed through the following formulation.

3 $$\begin{aligned} \begin{aligned} {}_{0}^{C}D_{t}^{\beta } e(t)&= \left[ -A\,e(t) + B\,f(e(t)) + C\,f(e(t-\tau (t))) + D u(t) \right] + h(t, e(t), e(t-\tau (t))) \frac{{dB}^{H}(t)}{dt}, \\ e(t_0 + l)&= \eta (l), \end{aligned} \end{aligned}$$

where $$e(t)=y(t)-x(t)$$, $$f(e(t))=f(y(t))-f(x(t))$$, $$f(e(t-\tau (t)))=f(y(t-\tau (t)))-f(x(t-\tau (t)))$$, and $$\eta (l)= \epsilon (l)-\delta (l)$$.

#### Control mechanism

To realize a mean square finite-time contractive synchronization between stochastic delayed neural networks (2) and the noise-free derive system (1), we design the following controller input *u*(*t*) is presented as:

4 $$\begin{aligned} u(t) =&u_1(t)+u_2(t) = -K (y(t)-x(t)) + \sum \limits _{k=0}^{\infty } [M_{k} (y(t)-x(t))-(y(t)-x(t))] \delta (t-t_k), \end{aligned}$$

where $K \in \mathbb {R}^{n \times n}$ is a constant matrix and $M_k$ is the impulsive control gain matrix; $\delta (\cdot )$ denotes the Dirac delta function; $t_0=0$ is the initial time, and $\{t_1, t_2, \ldots , t_{\mathscr {N}-1}, t_{\mathscr {N}}\}$ such that $t_{\mathscr {N}}<T$ for give $T>0$ is a finite sequence of impulsive instants with $\lim \limits _{k \rightarrow +\infty }t_k= +\infty ,$ where $\mathscr {N}$ denotes the number of impulse instances. Assume that throughout this paper the error signal $e(t)=y(t)-x(t)$ is right continuous at $t=t_k, k \in \mathbb {Z}_+,$ i.e., $e(t_k)=e(t_k^-).$ When $t\ne t_k, k \in \mathbb {Z}_+,$ according to (4), $u(t)=-K(y(t)-x(t)),$ then

5 $$\begin{aligned} {}_{0}^{C}D_{t}^{\beta } e(t)&= \left[ -A\,e(t) + B\,f(e(t)) + C\,f(e(t-\tau (t))) + D (-Ke(t)) \right] + h(t, e(t), e(t-\tau (t))) \frac{{dB}^{H}(t)}{dt}. \end{aligned}$$

When $t=t_k, k \in \mathbb {Z}_+$ by combining error (3) and control input (4), it is easy to obtain that form^25^

6 $$\begin{aligned} \Delta e(t_k)=e(t_k^+)-e(t_k)=D(M_k-1)e(t_k^-), \end{aligned}$$

where $$e(t_k^+)=\lim \limits _{h \rightarrow 0^+}e(t_k+h),$$ then

7 $$\begin{aligned} e(t_k^+)=DM_ke(t_k^-). \end{aligned}$$

The controller $u_2(t)$ induces instantaneous changes in the state of system (3) at the impulse instants $t_k$ that is, $u_2(t)$ acts as an impulsive control for system (3). Accordingly, the resulting closed-loop nonlinear delayed system under the hybrid control *u*(*t*) can be expressed as follows:

8 $$\begin{aligned} {\left\{ \begin{array}{ll} {}_{0}^{C}D_{t}^{\beta } e(t) = \left[ -(A+DK)\,e(t) + B\,f(e(t)) + C\,f(e(t-\tau (t))) \right] + h(t, e(t), e(t-\tau (t))) \dfrac{dB^{H}(t)}{dt}, t \ne t_k,\; k \in \mathbb {Z}_+, \\ e(t_k) = DM_{k}\, e(t_k^{-}), t = t_k, \\ e(t_0 + l) = \eta (l). \end{array}\right. } \end{aligned}$$

Hereafter, some necessary definitions and lemmas are presented in the following manner. They serve as essential preliminaries for the analysis and proofs developed later.

##### Definition 1

^46^ Let $\hslash _1$, $\hslash _2$, and *T* be positive real numbers with $\hslash _2 > \hslash _1$. The system (8) is said to achieve MSFTSn with respect to $(\hslash _1, \hslash _2, T)$ if $\sup \limits _{t \in [t_0-\tau ,\, t_0]} E\Vert \eta \Vert ^2 \le \hslash _1$ implies $E\Vert e(t)\Vert ^2 \le \hslash _2$, for all $t \in [t_0, T]$, where *E* denotes the expectation operator.

##### Definition 2

^43,46^ Assume that there exist positive constants $\hslash _1$, $\hslash _2$, $\hslash _3$, $\sigma$, and *T* with $\hslash _2> \hslash _1 > \hslash _3$, and $\sigma \in (t_0, T)$, then the systems (8) achieve MSFTCSn with respect to $(\hslash _1, \hslash _2, T)$, if it is MSFTSn and additionally satisfies $E\Vert e(t)\Vert ^2 \le \hslash _3$, for all $t \in [T-\sigma , T]$.

##### Definition 3

^50^ Let $\tfrac{1}{2}< H < 1$ be fixed. A real-valued standard fractional Brownian motion (fBm), $\{B^H(t),\, t \ge 0\}$, with Hurst parameter *H*, is a zero-mean Gaussian process possessing continuous sample paths. Its fundamental statistical properties are defined as

$$\begin{aligned} \mathbb {E}\{B^H(t)\}&= 0, \quad t \ge 0, \\ \mathbb {E}\{B^H(t)B^H(s)\}&= \tfrac{1}{2}\big (t^{2H} + s^{2H} - |t - s|^{2H}\big ). \end{aligned}$$

Here, *H* determines the self-similarity and long-range dependence of the process: when $H = 0.5$, $B^H(t)$ reduces to the standard Brownian motion, while $H > 0.5$ indicates positively correlated increments, implying a persistent stochastic influence.

##### Definition 4

^4^ A function $g \in C^{1} \left( [0,\infty ), \mathbb {R}\right)$ admits a fractional integral of order $\beta$ (with $0< \beta < 1$ and $t \ge t_0$), which is defined by

$$\begin{aligned} {}_{t_0} I_{t}^\beta g(t)=\frac{1}{\Gamma (\beta )} \int _{t_0}^t \frac{g(h)}{(t-h)^{1-\beta }} dh, \end{aligned}$$

where $\Gamma (\beta )$ refers to the Gamma function, given by $\Gamma (\beta )=\int \limits _0^{+\infty } e^{-s} s^{\beta -1} ds$.

##### Definition 5

^4^ Let $\beta \in (0,1)$ and $t \ge t_0$. For a function $g \in C^{1} \left( [0,\infty ), \mathbb {R}\right)$, the Caputo fractional derivative of order $\beta$ is given by

$$\begin{aligned} _{t_0}^C D_t^\beta g(t)=\frac{1}{\Gamma (1-\beta )} \int _{t_0}^t \frac{g^{\prime }(h)}{(t-h)^\beta } dh. \end{aligned}$$

##### Lemma 1

^4^ If $z \in C^1([0,+\infty ), \mathbb {R})$ *and* $n-1<\beta <n, \ \left( n \ge 1, n \in \mathbb {Z}_{+}\right)$*, then*

$$\begin{aligned} _{t_0} I_t^\beta \left( _{t_0}^C D_t^\beta z(t)\right) =z(t)-\sum _{k=0}^{n-1} \frac{t^k}{k!} z^{(k)}(0). \end{aligned}$$

*Under the condition* $0<\beta <1$*, the expression takes the simplified form:*

$$\begin{aligned} _{t_0} I_t^\beta \left( _{t_0}^C D_t^\beta z(t)\right) =z(t)-z(t_0) . \end{aligned}$$

##### Lemma 2

^11^ *Consider a continuously differentiable vector function* $y \in \mathbb {R}^n$*. For every* $t \ge t_0$ *and for all* $\beta \in (0,1)$*, the following inequality holds:*

$$\begin{aligned} {}_{t_0}^C D_t^\beta \left( y^T(t) \mathscr {P} y(t)\right) \le 2 y^T(t) \mathscr {P} {}_{t_0}^C D_t^\beta y(t), \end{aligned}$$

*where* $\mathscr {P} \in \mathbb {R}^{n \times n}$ *is a symmetric and positive definite matrix*.

##### Lemma 3

^12^ *Suppose* $f \in C^1 \left( [0,\infty ),\mathbb {R}\right)$ *is a function for which the Caputo fractional derivative satisfies*

$$\begin{aligned} _{t_0}^C D_t^{\beta } g(t) \le \theta g(t), \end{aligned}$$

*with* $\beta \in (0,1)$ *and* $\theta \in \mathbb {R}$*. Then the following estimate holds:*

$$\begin{aligned} g(t) \le g (t_0 ) \mathscr {E}_\beta \left( \theta (t-t_0)^\beta \right) , \end{aligned}$$

*where* $\mathscr {E}_{\beta }(\cdot )$ *denotes the Mittag–Leffler function, defined as* $\mathscr {E}_\beta (z)= \sum \limits _{k=0}^{\infty } \frac{z^{k}}{\Gamma (k\beta +1)}$.

##### Lemma 4

^24^ *For any vectors* $y, z \in \mathbb {R}^n$ *and any symmetric positive definite matrix* $\mathscr {Q} \in \mathbb {R}^{n \times n}$*, the following inequality holds:*

$$\begin{aligned} 2y^{T} z \le y^{T} \mathscr {Q}^{-1} y + z^{T} \mathscr {Q} z. \end{aligned}$$

##### Remark 1

The concepts of MSFTSn and MSFTCSn differ in their synchronization precision and robustness level. In MSFTSn, the synchronization error *e*(*t*) evolves from an initial region $\hat{h}_1$ to a smaller region $\hat{h}_2$ within a finite time, ensuring that the mean square error remains bounded thereafter. In contrast, MSFTCSn introduces an additional contractive condition that further confines the error within a tighter region $\hat{h}_3$ ($\hat{h}_3 < \hat{h}_2$) over $[T-\sigma , T]$, enforcing a continuous reduction of the synchronization error even after finite-time convergence. This contractive property guarantees higher synchronization precision and enhanced robustness against stochastic perturbations and delays. Hence, MSFTCSn represents a stronger and more reliable synchronization form, with its importance lying in its ability to maintain sustained stability and improved resilience compared to conventional MSFTSn.

## Main results

In this section, we establish the main results for MSFTSn and MSFTCSn for the FONNs considered. The analysis is carried out using an appropriate LF in combination with properties of the Mittag–Leffler function. These tools enable us to derive sufficient conditions that guarantee synchronization thoery under the given impulsive stochastic framework.

### NNs with continuous neuron activation function:

#### Theorem 1

*Let* $\{e(t)\}_{t \ge 0}$ *be a* $\mathscr {K}$*-valued stochastic process that represents a mild solution of (*8*) and* $w_{1}, w_{2} \in \mathfrak {K}$*. Assume that* $\hslash {1}$*,* $\hslash _{2}$*,* $\hslash _{3}$*,* $\sigma$*,* *T,* $\mu$*, and* $\rho$ *are positive constants satisfying* $\hslash _{2}> \hslash _{1} > \hslash _{3}$ *and* $\sigma \in (t_{0}, T)$*. Consider a locally Lipschitz continuous function* $V(t, e): \mathbb {R}{+} \times \mathbb {R}^{n} \rightarrow \mathbb {R}$*, where* $\mathscr {P}$ *and* $\hat{\mathscr {P}}$ *are positive definite matrices, and* $Q, G \in \mathbb {R}^{n \times n}$ *are arbitrary real matrices. Suppose that the following conditions hold:*

(i) $$\Phi =\begin{bmatrix} \Phi _{11} & \Phi _{12} \\ * & \Phi _{22} \end{bmatrix} < 0$$;
(ii) $$w_1 \ E\Vert e\Vert ^{2} \le EV(t, e) \le w_2 \ E\Vert e\Vert ^{2}$$;
(iii) $${}_{t_{0}}^{C} D_{t}^{\beta } E V(t, e(t)) \le \mu E V(t, e(t),$$$$t \ne t_{k}$$, $$k \in \mathbb {Z}_{+}$$;
(iv) $$E V(e(t)) \le \rho E V(e(t^{-}))$$, $$t = t_{k}$$;
(v) $$M_{k}^{T} \mathscr {P} M_{k} \le \rho \mathscr {P}$$;
(vi) $$\mathscr {P} D = D \hat{\mathscr {P}}$$;
(vii) The inequality
  9 $$\begin{aligned} \rho ^{ \left( \frac{T}{\vartheta _{2}} \right) } \left[ \mathscr {E}_{\beta }(\mu \ \vartheta _{2}^{\beta }) \right] ^{\left( \frac{T}{\vartheta _{1}} \right) +1} \le \frac{w_1 (\hslash _2)}{w_2 (\hslash _1)}, \forall t \in \left[ t_0, T \right] . \end{aligned}$$
  *Then, the NNs (*8*) achieve MSFTSn with respect to* $( \hslash _1, \hslash _2, T)$*. Furthermore, if the additional condition*
  $$\begin{aligned} \rho ^{ \left( \frac{T}{\vartheta _{2}} \right) } \left[ \mathscr {E}_{\beta }(\mu \ \vartheta _{2}^{\beta }) \right] ^{\left( \frac{T}{\vartheta _{1}} \right) +1} \le \frac{w_1 (\hslash _3)}{w_2 (\hslash _1)}, \forall t \in \left[ T-\sigma , T \right] . \end{aligned}$$
  *also holds, then the NNs (*8*) attain MSFTCSn with respect to* $(\hslash _{1}, \hslash _{2}, \hslash _{3}, \sigma , T)$.

*Where*

$$\begin{aligned} \Phi _{11}=&\begin{bmatrix} -\mathscr {P} A - A^{T} \mathscr {P} - D G- G^{T} D^{T} - \mathscr {L}_{1} \mathscr {M} & 0 & \mathscr {L}_{2} \mathscr {M} \\ * & - \mathscr {L}_{1} \mathscr {W} & 0 \\ * & * & -\mathscr {M} + \mathscr {Q} \\ \end{bmatrix}, \Phi _{12}=\begin{bmatrix} 0 & \mathscr {P} B & \mathscr {P} C \\ \mathscr {L}_2 \mathscr {W} & 0 & 0 \\ 0 & 0 & 0 \\ \end{bmatrix}, \\ \Phi _{22}=&\begin{bmatrix} -\mathscr {W}+\mathscr {Q} & 0 & 0 \\ * & -\mathscr {Q} & 0 \\ * & * & -\mathscr {Q} \\ \end{bmatrix}, \ \text {and} \ \text {control gain} \ K=\hat{\mathscr {P}}^{-1}G. \end{aligned}$$

#### Proof

To establish the synchronization criteria for NNs (8), we construct a suitable Lyapunov function candidate in quadratic form as follows:

10 $$\begin{aligned} V(e(t))= e^{T}(t) \mathscr {P} e(t), \end{aligned}$$

where $\mathscr {P}=\mathscr {P}^{T}$ is positive definite matrix. This quadratic structure is adopted because it ensures positive definiteness, captures the instantaneous energy of the synchronization error, and provides a convenient framework for deriving solvable LMI-based stability conditions. According to Lemma 2, when $t \ne t_{k}$ the fractional derivative of the Lyapunov function takes the following form:

11 $$\begin{aligned} {}_{t_0}^C D_t^\beta V(e(t))&\le 2 e^T(t) \mathscr {P} \, {}_{t_0}^C D_t^\beta e(t) \nonumber \\ &= 2 e^T(t) \mathscr {P} \left( \left[ -A\,e(t) + B\,f(e(t)) + C\,f(e(t-\tau (t))) - D K e(t) \right] + h(t, e(t), e(t-\tau (t))) \frac{{dB}^{H}(t)}{dt} \right) \nonumber \\&= -2 e^T(t) \mathscr {P} A e(t) + 2 e^T(t) \mathscr {P} B f(e(t)) + 2 e^T(t) \mathscr {P} C f(e(t-\tau (t))) - 2 e^T(t) \mathscr {P} D K e(t) \nonumber \\ &\quad + 2 e^T(t) \mathscr {P} h(t, e(t), e(t-\tau (t))) \frac{{dB}^{H}(t)}{dt}. \end{aligned}$$

Hereafter, the cross-product and coupling terms that arise in the fractional derivative of *V*(*e*(*t*)) are handled using Lemma 2.4. This inequality decouples the product terms and converts them into diagonal quadratic forms suitable for the LMI formulation, from which the following relations can be derived based on Lemma 2.4.

12 $$\begin{aligned}&2 e^{T}(t) \mathscr {P} B f(e(t)) \le e^{T}(t) \mathscr {P} B Q^{-1} B^{T}\mathscr {P} e(t) + f^{T}(e(t)) Q f(e(t)), \end{aligned}$$

13 $$\begin{aligned}&2 e^T(t) \mathscr {P} C f(e(t-\tau (t))) \le e^{T}(t) \mathscr {P} C Q^{-1} C^{T} \mathscr {P} e(t) + f^{T}(e(t-\tau (t))) Q f(e(t-\tau (t))). \end{aligned}$$

Taking Assumption 1 into account, the following inequality can be derived implies that

$$\begin{aligned}&(f(e(t))-\mathscr {L}_{1} \ e(t) )(f(e(t))-\mathscr {L}_{2} \ e(t) ) \le 0, \\&(f(e(t-\tau (t)))-\mathscr {L}_{1} \ e(t-\tau (t)) )(f(e(t-\tau (t)))-\mathscr {L}_{2} \ e(t-\tau (t)) ) \le 0, \end{aligned}$$

where $\mathscr {L}_{1}$ and $\mathscr {L}_{2}$ are Lipschitz matrices. Let $\mathscr {M}$ and $\mathscr {W}$ be $n \times n$ diagonal matrices. Then, by applying the above inequality, we obtain

14 $$\begin{aligned}&\begin{pmatrix} e(t) \\ f(e(t)) \\ \end{pmatrix}^{T} \begin{pmatrix} -\mathscr {L}_1 \mathscr {M} & \mathscr {L}_{2}\mathscr {M} \\ * & -\mathscr {M} \end{pmatrix} \begin{pmatrix} e(t) \\ f(e(t)) \end{pmatrix} \ge 0, \end{aligned}$$

15 $$\begin{aligned}&\begin{pmatrix} e(t-\tau (t)) \\ f(e(t-\tau (t))) \end{pmatrix}^{T} \begin{pmatrix} -\mathscr {L}_{1}\mathscr {W} & \mathscr {L}_{2}\mathscr {W} \\ * & -\mathscr {W} \end{pmatrix} \begin{pmatrix} e(t-\tau (t)) \\ f(e(t-\tau (t))) \end{pmatrix} \ge 0. \end{aligned}$$

By substituting (12)-(15) along with condition (ii) into (11), one can get

16 $$\begin{aligned} {}_{t_0}^C D_t^\beta V(e(t)) \le&- e^{T}(t)(\mathscr {P} A + A^{T} \mathscr {P}) e(t)+ e^{T}(t) \mathscr {P} B Q^{-1} B^{T}\mathscr {P} e(t) + f^{T}(e(t)) Q f(e(t))+ e^{T}(t) \mathscr {P} C Q^{-1} C^{T} \mathscr {P} e(t) \nonumber \\&+ f^{T}(e(t-\tau (t))) Q f(e(t-\tau (t))) - 2 e^T(t) \mathscr {P} D K e(t) +\begin{pmatrix} e(t) \\ f(e(t)) \\ \end{pmatrix}^{T} \begin{pmatrix} -\mathscr {L}_1 \mathscr {M} & \mathscr {L}_{2}\mathscr {M} \\ * & -\mathscr {M} \end{pmatrix} \begin{pmatrix} e(t) \\ f(e(t)) \end{pmatrix} \nonumber \\&+\begin{pmatrix} e(t-\tau (t)) \\ f(e(t-\tau (t))) \end{pmatrix}^{T} \begin{pmatrix} -\mathscr {L}_{1}\mathscr {W} & \mathscr {L}_{2}\mathscr {W} \\ * & -\mathscr {W} \end{pmatrix} \begin{pmatrix} e(t-\tau (t)) \\ f(e(t-\tau (t))) \end{pmatrix}+ 2 e^T(t) \mathscr {P} h(t, e(t), e(t-\tau (t))) \frac{{dB}^{H}(t)}{dt} \nonumber \\ =&\xi ^{T}(t) \Delta \xi (t) + \mu e^{T}(t) \mathscr {P} e(t) + 2 e^T(t) \mathscr {P} h(t, e(t), e(t-\tau (t))) \frac{{dB}^{H}(t)}{dt} \nonumber \\ \le&\mu V(e(t)) + 2 e^T(t) \mathscr {P} h(t, e(t), e(t-\tau (t))) \frac{{dB}^{H}(t)}{dt}, \end{aligned}$$

where $$\xi (t) = (e^{T} (t),e^{T}$$$$(t - \tau (t)),$$$$f^{T} (e(t)),f^{T} (e(t - \tau (t))))^{T} ,\;$$$$\Delta =\begin{bmatrix} \Delta _{11} & 0 & \mathscr {L}_{2} \mathscr {M} & 0 \\ * & - \mathscr {L}_{1} \mathscr {W} & 0 & \mathscr {L}_{2} \mathscr {W} \\ * & * & -\mathscr {M} + \mathscr {Q} & 0 \\ * & * & * & -\mathscr {W} + \mathscr {Q} \end{bmatrix}$$, and

17 $$\begin{aligned} \Delta _{11}= -\mathscr {P} (A+DK) - (A+DK)^{T} \mathscr {P} - \mathscr {L}_{1} \mathscr {M} + \mathscr {P} B Q^{-1} B^{T}\mathscr {P} + \mathscr {P} C Q^{-1} C^{T} \mathscr {P}. \end{aligned}$$

By employing the condition (vi), we rewrite the LMI term

18 $$\begin{aligned} \Delta _{11}=-\mathscr {P}A-DG-A^T\mathscr {P}^T-D^TG^T- \mathscr {L}_{1} \mathscr {M} + \mathscr {P} B Q^{-1} B^{T}\mathscr {P} + \mathscr {P} C Q^{-1} C^{T} \mathscr {P}, \end{aligned}$$

with $K=\hat{\mathscr {P}}^{-1}G$. Moreover, by taking the Schur complement of the matrix $\Delta$, we obtain the matrix $\Phi$. According to condition (i) of Theorem 1, it then follows that $\Delta < 0$. Applying the expectation operator to both sides of (16) and invoking Definition 3, the following relation is derived:

19 $$\begin{aligned} E \left[ {}_{t_0}^C D_t^\beta V(e(t)) \right] \le \mu \ E V(e(t)). \end{aligned}$$

After that, applying the fractional integral to both sides of (19) and using Lemma 3, we obtain

20 $$\begin{aligned} {}_{t_0} I_t^\beta \left[ {}_{t_0}^C D_t^\beta EV(e(t)) \right] \le&\mu \ {}_{t_0} I_t^\beta [E V(e(t))] \nonumber \\ E V(e(t)) \le&E V(e(t_{0}))+\frac{\mu }{\Gamma (\beta )} \int _{t_0}^{t} E V(e(s)) (t-s)^{\beta -1} ds \nonumber \\ E V(e(t)) \le&E V(e(t_{0})) \ \mathscr {E}_{\beta }(\mu (t-t_{0})^{\beta }). \end{aligned}$$

Let $t = t_{k}$; in this case, condition (v) yields

21 $$\begin{aligned} V(e(t_{k}))&= e^T(t_{k}) \mathscr {P} e(t_{k}) \nonumber \\&= e^{T}(t_{k}^{-}) M_{k}^{T} \mathscr {P} M_{k} e(t_{k}^{-}) \nonumber \\&\le e^{T}(t_{k}^{-}) \rho \mathscr {P} e(t_{k}^{-}) = \rho V(e(t_k^{-})). \end{aligned}$$

Substituting inequality (21) into inequality (20), we have

$$\begin{aligned} E V(e(t_{k})) \le&\ \rho \ \mathscr {E}_{\beta }(\mu (t-t_{k-1})^{\beta }) \ E V(e(t_{k-1})). \end{aligned}$$

Repeating the above inequality for $k=\{1,2,\dots , \mathscr {N}\}$, which yields

$$\begin{aligned} E V(e(t_{\mathscr {N}})) \le \rho ^{\mathscr {N}} \left( \prod \limits _{i=1}^{m} \mathscr {E}_{\beta }(\mu (t_{i}-t_{i-1})^{\beta }) \right) \ E V(e(t_{0})), \end{aligned}$$

then for any $t \in [t_{\mathscr {N}}, t_{\mathscr {N}+1})$,

$$\begin{aligned} E V(e(t)) \le&\mathscr {E}_{\beta }(\mu (t-t_{\mathscr {N}})^{\beta }) \ E V(e(t_{\mathscr {N}})) \\ \le&\rho ^{\mathscr {N}} \mathscr {E}_{\beta }(\mu (t-t_{\mathscr {N}})^{\beta }) \left( \prod \limits _{i=1}^{m} \mathscr {E}_{\beta }(\mu (t_{i}-t_{i-1})^{\beta }) \right) \ E V(e(t_{0})). \end{aligned}$$

Based on the impulse sequence condition, one can get

$$\begin{aligned} E V(e(t)) \le \rho ^{ \left( \frac{t}{\vartheta _{2}} \right) } \left[ \mathscr {E}_{\beta }(\mu \ \vartheta _{2}^{\beta }) \right] ^{\left( \frac{t}{\vartheta _{1}} \right) +1} \ E V(e(t_{0})). \end{aligned}$$

Therefore, for $t \in [0, T]$ such that

$$\begin{aligned} E V(e(t)) \le \rho ^{ \left( \frac{T}{\vartheta _{2}} \right) } \left[ \mathscr {E}_{\beta }(\mu \ \vartheta _{2}^{\beta }) \right] ^{\left( \frac{T}{\vartheta _{1}} \right) +1} \ E V(e(t_{0})). \end{aligned}$$

Using condition (ii), we have

22 $$\begin{aligned} w_1E ||e(t)||^{2} \le E V(e(t)) \le&\rho ^{ \left( \frac{T}{\vartheta _{2}} \right) } \left[ \mathscr {E}_{\beta }(\mu \ \vartheta _{2}^{\beta }) \right] ^{\left( \frac{T}{\vartheta _{1}} \right) +1} \ w_2 E ||\eta ||^{2} \nonumber \\&\le \rho ^{ \left( \frac{T}{\vartheta _{2}} \right) } \left[ \mathscr {E}_{\beta }(\mu \ \vartheta _{2}^{\beta }) \right] ^{\left( \frac{T}{\vartheta _{1}} \right) +1} \ w_2(\hslash _1) \le w_1(\hslash _2), \quad t \in [t_0, T]. \end{aligned}$$

From inequality (22), it follows that $E\Vert e(t)\Vert ^{2} \le \hslash _{2}$, which guarantees that the error dynamics meets the requirements of Definition 1 and the first inequality in condition (v). Therefore, neural networks (8) can be regarded as achieving mean square finite-time synchronization with respect to the parameters $(\hslash _{1}, \hslash _{2}, T)$. In addition, it satisfies

23 $$\begin{aligned} w_1E ||e(t)||^{2}&\le E V(e(t)) \le \rho ^{ \left( \frac{T}{\vartheta _{2}} \right) } \left[ \mathscr {E}_{\beta }(\mu \ \vartheta _{2}^{\beta }) \right] ^{\left( \frac{T}{\vartheta _{1}} \right) +1} \ w_2 E ||\eta ||^{2} \nonumber \\&\le \rho ^{ \left( \frac{T}{\vartheta _{2}} \right) } \left[ \mathscr {E}_{\beta }(\mu \ \vartheta _{2}^{\beta }) \right] ^{\left( \frac{T}{\vartheta _{1}} \right) +1} \ w_2(\hslash _1) \le w_1(\hslash _3), \quad t \in [T-\sigma , T]. \end{aligned}$$

By inequality (23), one obtains $E||e(t)||^{2} \le \hslash _{3}$, which directly confirms that system (8) attains MSFTCSn under Definition 2 and the second inequality in condition (v) with parameters $(\hslash _{1}, \hslash _{2}, \hslash _{3}, \sigma , T)$. The proof is completed. $\square$

#### Remark 2

The set of conditions (*i*)–(*vii*) is fundamental in guaranteeing the finite-time synchronization properties of the considered stochastic delayed neural networks with impulses. Condition (*i*) enforces the negativity of the constructed block matrix $\Phi$, which is the core feasibility requirement for the LMI framework. Conditions (*ii*) and (*iii*) restrict the Lyapunov functional growth by relating it to the error terms through the weighting functions and the $\mu$-term, thereby ensuring boundedness and decay of trajectories. Condition (*iv*) controls the impulsive effects by constraining the jump behavior of the Lyapunov functional, while condition (*v*) ensures that the impulse matrices preserve stability by limiting their interaction with the Lyapunov matrix. Condition (*vi*) guarantees consistency between the system matrices, and condition (*vii*) provides the inequality that explicitly links the weighting functions with the finite-time bound. Together, these conditions form a tight and nonconservative set of criteria that directly ensure MSFTSn and, under the additional inequality, extend the results to MSFTCSn. It is worth emphasizing that all the conditions are very important in combination, as they are not mere restrictions but necessary requirements to achieve the desired finite-time synchronization behavior.

#### Remark 3

Theorem 3.1 provides the theoretical basis for deriving the corresponding feedback control gain matrix *K* from the proposed LMI-based stability conditions. Specifically, the feedback gain matrix *K* is determined from the LMI conditions (i) and (vi), which guarantee the negativity of the symmetric matrix $\Phi$ and ensure compatibility among the positive definite matrices that define the Lyapunov functional used to analyze the synchronization of the delayed fractional-order neural network system (8). The matrix term involving *K* in the LMI formulation is expressed in (17). Here, the matrix *D* is assumed to be a full-rank column matrix, implying that both *D* and $\mathscr {P}D$ are linearly independent for any $\mathscr {P} > 0$. Hence, a nonsingular matrix $\hat{\mathscr {P}}$ is defined such that the equality condition (vi) is satisfied for some $\mathscr {P} > 0$. If condition (vi) holds for a positive definite $\mathscr {P}$, then $\hat{\mathscr {P}}$ must also be nonsingular. This equality constraint serves as an essential condition in reformulating the LMI problem as a feasibility problem. To transform the original non-convex constraint into a convex form, the procedure described in^51,52^ is employed, where the equality condition (vi) is replaced by the following LMI with a small scalar $\nu > 0$:

$$\begin{aligned} \begin{bmatrix} -\nu I & \mathscr {P}D - D\hat{\mathscr {P}} \\ * & -\nu I \end{bmatrix} < 0. \end{aligned}$$

By introducing an auxiliary variable $G = \hat{\mathscr {P}}K$ to remove the bilinear product between $\mathscr {P}$ and *K*, the expression of $\Delta _{11}$ can be rewritten in a convex form as in (18 ) and the corresponding feedback control gain is explicitly obtained as $K = \hat{\mathscr {P}}^{-1}G.$ This formulation ensures that the resulting LMI constraints are convex and computationally tractable, allowing the control gain matrix *K* to be systematically computed from the feasible solution of the proposed LMI conditions.

### NNs with discontinuous neuron activation function:

The analysis of MSFTSn and MSFTCSn for fractional-order stochastic neural networks with discontinuous neuron activation functions is highly challenging. Discontinuities, arising from switching or saturation effects, hinder the direct use of classical smooth-function techniques. When combined with stochastic disturbances and fractional-order memory effects, the system dynamics become even more complex. To overcome these issues, set-valued map theory provides a rigorous framework for establishing synchronization criteria in such networks.

We proceed by considering the neural networks (8), in which the neuron activation functions are assumed to be discontinuous:

24 $$\begin{aligned} \begin{aligned} {}_{0}^{C}D_{t}^{\beta } e(t)&= \left[ -A \ e(t) + B \ f(e(t)) + C \ f(e(t -\tau (t)))-D K e(t) \right] + h(t, e(t), e(t-\tau (t))) \frac{{dB}^{H}(t)}{dt}, \quad t \ne t_k, \quad k \in \mathbb {Z}_+, \\ e(t_k)&= DM_{k}\, e(t_k^{-}), t = t_k, \\ e(t_0 + l)&= \eta (l). \end{aligned} \end{aligned}$$

The nonlinear function $f(\cdot ) : \mathbb {R}^{n} \rightarrow \mathbb {R}^{n}$ is assumed to be locally bounded and Lebesgue measurable, while possibly exhibiting discontinuities at certain points $e(\cdot )$. In such situations, the solutions of system (24) are formulated within the framework of Filippov regularization, where the corresponding set-valued map of $f(\cdot )$ is defined in the sense of Filippov as follows:

$$\begin{aligned} \gamma (e(t), e(t-\tau (t))) = \bigcap _{\begin{array}{c} r>0 \\ r>0 \end{array}} \bigcap _{\begin{array}{c} mes(L)=0 \\ mes(K)=0 \end{array}} \overline{co} \left[ \ f(\mathbb {B}(e(t), r)/L, \mathbb {B}(e(t-\tau (t)), r)/K ) \right] , \end{aligned}$$

where *mes*(*L*) and *mes*(*K*) represents the Lebesgue measure of the set *L* and *K*, respectively; The notation $\mathbb {B}(e(t), r)=\{z: ||z-e(t)|| \le r_1 \}$ with center *e*(*t*) and radius *r* depicts a ball; In a similar way, $\mathbb {B}(e(t-\tau (t)), r)$ also depicts a ball with center $e(t-\tau (t))$ and radius *r*.

#### Definition 6

^28^ The state *e*(*t*) is referred to as a Filippov solution of the discontinuous neural networks (24) if, for any interval $[t_{k}, t_{k+1}) \subseteq \mathbb {I}$, it is absolutely continuous and satisfies the following fractional-order stochastic time-delayed impulsive inclusion (FOSTDII):

$$\begin{aligned} {\left\{ \begin{array}{ll} {}_{0}^{C}D_{t}^{\beta } e(t) \in \left[ -A \ e(t) + B \ \overline{co}[f(e(t))] + C \ \overline{co}[f(e(t - \tau (t)))] -D K e(t) \right] + h(t, e(t), e(t-\tau (t))) \frac{{dB}^{H}(t)}{dt}, \quad t \ne t_k, \\ e(t_k) = DM_{k}\, e(t_k^{-}), t = t_k, \\ e(t_0 + l) = \eta (l). \end{array}\right. } \end{aligned}$$

By the local boundedness of *f*, the set-valued map $\gamma$ is nonempty, compact, convex and USC. In addition, the existence of local solution of $e(t_{0}, e_{t_{0}})(t)$ can be guaranteed for any $(t_{0}, e_{t_0}) \in \mathbb {R}_{+} \times \mathbb {R}^{n}$. If *e*(*t*) is regarded as a solution of the above FOSTDII, then there exists a measurable function $\gamma (t) \in \overline{co}[f(e(\cdot ))]$, such that

25 $$\begin{aligned} {\left\{ \begin{array}{ll} {}_{0}^{C}D_{t}^{\beta } e(t) = \left[ -A \ e(t) + B \ \gamma (e(t)) + C \ \gamma (e(t-\tau (t))) -D K e(t) \right] + h(t, e(t), e(t-\tau (t))) \frac{{dB}^{H}(t)}{dt}, \quad t \ne t_k, \\ e(t_k) = DM_{k}\, e(t_k^{-}), t = t_k, \\ e(t_0 + l) = \eta (l). \end{array}\right. } \end{aligned}$$

Within this framework, we proceed to derive theoretical synchronization criteria for NNs characterized by discontinuous activation functions.

#### Theorem 2

*Assume that conditions (ii)–(iv) and (vi) of Theorem* 1*are satisfied. Let* $\mathscr {Z}, H, G \in \mathbb {R}^{n \times n}$ *be arbitrary real matrices, and* $\mathscr {P}$*,* $\hat{\mathscr {P}}$ *are positive definite matrices. Let* $\hslash _{1}, \hslash _{2}, \hslash _{3}, \sigma$*, and* *T* *be positive constants such that* $\hslash _{2}> \hslash _{1} > \hslash _{3}$ *and* $\sigma \in (t_{0}, T)$*. Assume* $w_{1}, w_{2} \in \mathfrak {K}$*,* $\omega , \alpha , \nu _{1}, \nu _{4}$ *are positive constants and* $\nu _{2}, \nu _{3}$ *are negative constants. Suppose the following conditions are satisfied:*

(i) $$\mathbb {X}=\begin{bmatrix} \mathbb {X}_{11} & \nu _{1} \mathscr {Z} B -\nu _{2} A^{T} \mathscr {Z} & \nu _{1} \mathscr {Z} C -\nu _{3} A^{T} \mathscr {Z} & -\nu _{1}\mathscr {Z} -\nu _{4} A^{T} \mathscr {Z} & \mathscr {P}B & \mathscr {P}C \\ * & H + \nu _{2} \mathscr {Z} B & \nu _{2}\mathscr {Z} C+\nu _{3}B^{T}\mathscr {Z} & -\nu _{2}\mathscr {Z}+\nu _{4} B^{T} \mathscr {Z} & 0 & 0 \\ * & * & H + \nu _{3} \mathscr {Z} C & -\nu _{3} \mathscr {Z}+\nu _{4} B^{T} \mathscr {Z} & 0 & 0\\ * & * & * & -\nu _{4} \mathscr {Z} & 0 & 0 \\ * & * & * & * & -H & 0 \\ * & * & * & * & 0 & -H \end{bmatrix} < 0$$*; where* $$\mathbb {X}_{11}=-\mathscr {P} A- A^{T} \mathscr {P}- D G - G^{T} D^{T} -\nu _{1} \mathscr {Z} A - \omega \mathscr {P}$$*, and* $$K=\hat{\mathscr {P}}^{-1}G$$.
(ii) $$M_{k}^{T} \mathscr {P} M_{k} \le \alpha \mathscr {P}$$*;*
(iii) *The inequality*
  26 $$\begin{aligned} \alpha ^{ \left( \frac{T}{\vartheta _{2}} \right) } \left[ \mathscr {E}_{\beta }(\omega \ \vartheta _{2}^{\beta }) \right] ^{\left( \frac{T}{\vartheta _{1}} \right) +1} \le \frac{w_1 (\hslash _2)}{w_2 (\hslash _1)}, \forall t \in \left[ t_0, T \right] . \end{aligned}$$
  *Then, the NNs (*8*) achieve MSFTSn with respect to* $( \hslash _1, \hslash _2, T)$*. Furthermore, if the additional condition*
  $$\begin{aligned} \alpha ^{ \left( \frac{T}{\vartheta _{2}} \right) } \left[ \mathscr {E}_{\beta }(\omega \ \vartheta _{2}^{\beta }) \right] ^{\left( \frac{T}{\vartheta _{1}} \right) +1} \le \frac{w_1 (\hslash _3)}{w_2 (\hslash _1)}, \forall t \in \left[ T-\sigma , T \right] , \end{aligned}$$
  *also holds, then the NNs (*8*) attain MSFTCSn with respect to* $(\hslash _{1}, \hslash _{2}, \hslash _{3}, \sigma , T)$.

#### Proof

Consider the Lyapunov function $V(e(t))= e^{T}(t) \mathscr {P} e(t)$, where $\mathscr {P}$ is positive definite matrix. From Lemma 2, the fractional derivative of *V*(*t*) for $t \ne t_{k}$ is expressed as:

27 $$\begin{aligned} {}_{t_0}^C D_t^\beta V(e(t))&\le 2 e^T(t) \mathscr {P} \, {}_{t_0}^C D_t^\beta e(t) \nonumber \\ &= 2 e^T(t) \mathscr {P} \left( \left[ -A\,e(t) + B\,\gamma (e(t)) + C\,\gamma (e(t-\tau (t))) - D K e(t) \right] + h(t, e(t), e(t-\tau (t))) \frac{{dB}^{H}(t)}{dt} \right) \nonumber \\&= -2 e^T(t) \mathscr {P} A e(t) + 2 e^T(t) \mathscr {P} B \gamma (e(t)) + 2 e^T(t) \mathscr {P} C \gamma (e(t-\tau (t))) - 2 e^T(t) \mathscr {P} D K e(t) \nonumber \\ &\quad + 2 e^T(t) \mathscr {P} h(t, e(t), e(t-\tau (t))) \frac{{dB}^{H}(t)}{dt}. \end{aligned}$$

Following Lemma 4, one can get

28 $$\begin{aligned}&2 e^{T}(t) \mathscr {P} B \gamma (e(t)) \le e^{T}(t) \mathscr {P} B H^{-1} B^{T}\mathscr {P} e(t) + \gamma ^{T}(e(t)) H \gamma (e(t)), \end{aligned}$$

29 $$\begin{aligned}&2 e^T(t) \mathscr {P} C \gamma (e(t-\tau (t))) \le e^{T}(t) \mathscr {P} C H^{-1} C^{T} \mathscr {P} e(t) + \gamma ^{T}(e(t-\tau (t))) H \gamma (e(t-\tau (t))). \end{aligned}$$

Combining (28) and (29) with (27), it follows that

30 $$\begin{aligned} {}_{t_0}^C D_t^\beta V(e(t)) \le&- e^{T}(t)(\mathscr {P} A + A^{T} \mathscr {P}) e(t)+ e^{T}(t) \mathscr {P} B H^{-1} B^{T}\mathscr {P} e(t) + \gamma ^{T}(e(t)) H \gamma (e(t))+ e^{T}(t) \mathscr {P} C H^{-1} C^{T} \mathscr {P} e(t) \nonumber \\ &+ \gamma ^{T}(e(t-\tau (t))) H \gamma (e(t-\tau (t))) - 2 e^T(t) \mathscr {P} D K e(t) + 2 e^T(t) \mathscr {P} h(t, e(t), e(t-\tau (t))) \frac{{dB}^{H}(t)}{dt}. \end{aligned}$$

In the sequel, the derivation proceeds by incorporating the free-weighting matrix methodology into the following formulation. Since the Lipschitz continuity condition adopted in the continuous case cannot be applied under discontinuous dynamics, the free-weighting matrix technique is introduced to flexibly handle the coupling between the error terms and their delayed components. This approach provides additional degrees of freedom in the Lyapunov analysis and ensures the feasibility of the LMI-based synchronization conditions for the discontinuous case.

31 $$\begin{aligned} {[}\nu _{1} e^{T}(t)+\nu _{2} \gamma ^{T}(e(t))+\nu _{3} \gamma ^{T}(e(t-\tau (t)))+\nu _{4} ({}_{t_{0}}^{C} \textsf{D}_{t}^{q} e(t))^{T}] \mathscr {Z} [-A \ e(t) +B \ \gamma (e(t))+C \ \gamma (e(t-\tau (t))) - {}_{t_{0}}^{C} \textsf{D}_{t}^{\beta } e(t)]=0, \end{aligned}$$

where $\nu _{1}, \nu _{2}, \nu _{3},$ and $\nu _{4}$ are constant parameters, and $\mathscr {Z}$ denotes an arbitrary real matrix. By substituting (31) into (30), the following expression is obtained:

32 $$\begin{aligned} {}_{t_0}^C D_t^\beta V(e(t)) \le \varphi ^{T}(t) \Pi \varphi (t) + \omega e^{T} P e(t) + 2 e^T(t) \mathscr {P} h(t, e(t), e(t-\tau (t))) \frac{{dB}^{H}(t)}{dt}, \end{aligned}$$

where $$\gamma (t)=(e^{T}(t), \ \gamma ^{T}(e(t)),$$$$\ \gamma ^{T}(t-\tau (t)), \ {}_{t_0}^C D_t^\beta e^{T}(t) )^{T}$$, $$\Pi =\begin{bmatrix} \Pi _{11} & \Pi _{12} & \Pi _{13} & -\nu _{1}\mathscr {Z} -\nu _{4} A^{T} \mathscr {Z} \\ * & H + \nu _{2} \mathscr {Z} B & \nu _{2}\mathscr {Z} C+\nu _{3} B^{T} \mathscr {Z} & -\nu _{2}\mathscr {Z}+\nu _{4} B^{T} \mathscr {Z} \\ * & * & H + \nu _{3} \mathscr {Z} C & -\nu _{3} \mathscr {Z}+\nu _{4} C^{T} \mathscr {Z} \\ * & * & * & -\nu _{4} \mathscr {Z} \end{bmatrix}$$, and $$\Pi _{{11}} = - {\mathcal{P}}A - A^{T} {\mathcal{P}}$$$$+ {\mathcal{P}}BH^{{ - 1}} B^{T} {\mathcal{P}}$$$$+ {\mathcal{P}}CH^{{ - 1}} C^{T} {\mathcal{P}} - 2{\mathcal{P}}DK - \nu _{1} {\mathcal{Z}}A - \omega {\mathcal{P}}$$, $$\Pi _{12}=\nu _{1} \mathscr {Z} B -\nu _{2} A^{T} \mathscr {Z}$$, $$\Pi _{13}=\nu _{1} \mathscr {Z} C -\nu _{3} A^{T} \mathscr {Z}$$. Applying the Schur complement to $$\Pi$$ leads to the matrix $$\mathbb {X}$$. Condition (i) of Theorem 2 guarantees that $$\Pi < 0$$. Therefore, applying the expectation operator to both sides of (32) in accordance with Definition 3 yields the following result:

$$\begin{aligned} E \left[ {}_{t_0}^C D_t^\beta V(e(t)) \right] \le \omega \ E V(e(t)). \end{aligned}$$

From the above inequality, it is evident that the structure coincides with inequality (19) in Theorem 1. Therefore, by following the procedure of Theorem 1, the intermediate derivations are omitted. In view of conditions (ii) and (iii) of Theorem 2, the criteria for MFTSn and MFTCSn of the FOTDSII (25) are established with respect to the parameters $(\hslash _{1}, \hslash _{2}, \hslash _{3}, \sigma , T)$. This completes the proof. $\square$

#### Remark 4

Earlier investigations^15,16^, and^29^ have explored finite-time synchronization of FODNNs using single-action control strategies such as fractional feedback, impulsive, and matrix projection methods. Although these approaches achieved synchronization under delays and uncertainties, they mainly focused on deterministic or partially uncertain systems without addressing stochastic effects or contractive synchronization properties. Likewise, studies^46–48^ examined MSFTS and MSFTSn of impulsive and stochastic systems through Lyapunov inequalities and stochastic differential techniques, but these efforts were mostly limited to integer-order dynamics and lacked consideration of hybrid control structures. To bridge these gaps, the present research develops a unified framework for MSFTCSn in FOSDNNs, applicable to both continuous and discontinuous control cases. The proposed hybrid control mechanism combines continuous feedback with impulsive regulation, exploiting the memory property of fractional derivatives to enhance damping, transient smoothness, and disturbance suppression. Through fractional Lyapunov functionals and impulsive differential inequalities, rigorous finite-time contractive conditions are derived under stochastic perturbations and delays, ensuring robust synchronization. This formulation achieves faster convergence, improved robustness, and greater control efficiency while extending applicability to a wider class of hybrid fractional-order systems.

## Numerical simulation

### Example 1

$$\underline{{{\textbf {Continuous case:}}}}$$

The neural networks described by equation (1) utilize the following parameter matrices:

$$\begin{aligned} A=\begin{bmatrix} 1.3 & 0 \\ 0 & 1.3 \end{bmatrix}, B=\begin{bmatrix} 1+\pi /4 & 20 \\ 0.1 & 1+\pi /4 \end{bmatrix}, C=\begin{bmatrix} -1.3\sqrt{(}3) \ \pi /4 & 0.1 \\ 0.1 & -1.3\sqrt{(}3) \ \pi /4 \end{bmatrix}, D=\begin{bmatrix} 1 & 0 \\ 0 & 1 \end{bmatrix}. \end{aligned}$$

The neuron activation function is defined as $f(x(\cdot ))=\frac{1}{2}(|x(\cdot )+1|-|x(\cdot )-1|)$ where the corresponding Lipschitz matrices are chosen as $\mathscr {L}_{1}=diag(0.6, 0.4)$ and $\mathscr {L}_{2}=diag(0.3, 0.1)$. In addition, the matrix $\mathscr {Q}$ is specified as *diag*(0.53, 0.52). The fractional order is set to $\beta =0.95$ and the time delay is defined by $\tau (t)=\frac{e^{t}}{e^{t}+1}$.

**Fig. 1:**
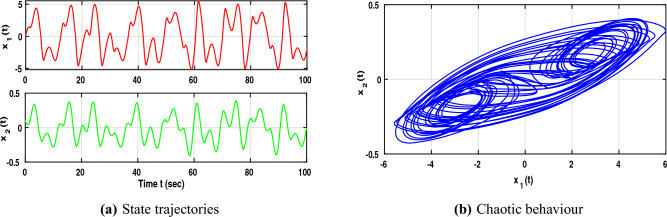
Dynamic behaviour of NNs (1).

With the chosen parameter settings, Fig. 1a illustrates the time evolution of the NN states, where the irregular and non-periodic oscillations reflect the complex dynamics introduced by time delays. Meanwhile, Fig. 1b depicts the phase trajectories of the time-delayed NNs (1), confirming the presence of chaotic motion characterized by high sensitivity to initial conditions and delay-induced nonlinear responses. Following this, consider the impulse input $$M_{1}=\begin{bmatrix} 0.8 & 0.0 \\ 0.31 & 0.01 \end{bmatrix}$$. The corresponding controller design procedure can now be systematically formulated using the LMI-based algorithm described below. Based on these parameter values, the subsequent feasibility solution can be derived using the MATLAB LMI Toolbox:

**Algorithm:**
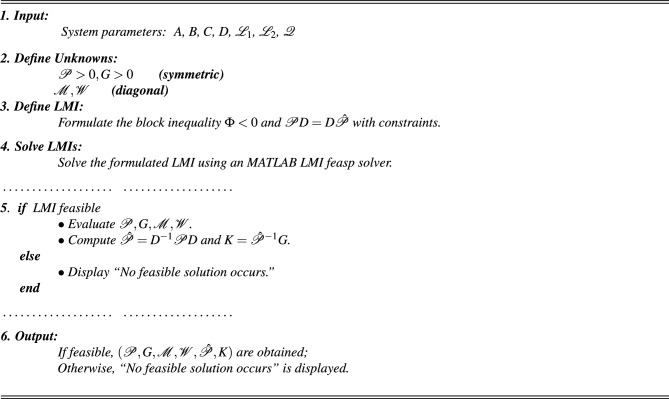
LMI-Based controller design.

**Fig. 2:**
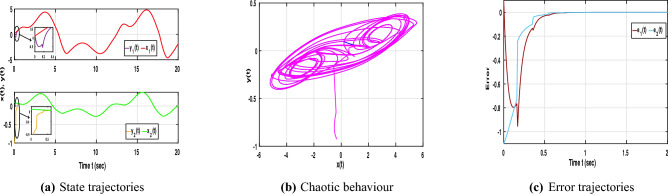
Dynamic behaviour of NNs (1) and (2) without stochastic influence with control input.

Following the aforementioned LMI-based algorithm, the feasibility solution corresponding to the selected parameter values can be obtained using the MATLAB LMI Toolbox:

$$\begin{aligned} \mathscr {P}=\begin{bmatrix} 0.0104 & -0.0176 \\ -0.0176 & 0.2088 \end{bmatrix}, \mathscr {M}=\begin{bmatrix} 1.1201 & 0 \\ 0 & 1.1201 \end{bmatrix}, \mathscr {W}=\begin{bmatrix} 1.2063 & 0 \\ 0 & 1.2063 \end{bmatrix}, G=\begin{bmatrix} 0.1584 & 0.0135 \\ 0.0135 & 0.0364 \end{bmatrix}, \end{aligned}$$

and the control gain *K* is obtained as $$K=\begin{bmatrix} 17.9387 & 1.8622 \\ 1.5788 & 0.3316 \end{bmatrix}$$. Under the designed control gain and impulse effects, Fig. 2a shows that the slave system trajectories quickly follow those of the master system, indicating effective control action.

As illustrated in Fig. 2b, the phase portraits of both networks nearly overlap, confirming successful synchronization. Meanwhile, Fig. 2c depicts the error trajectories converging rapidly to zero, demonstrating that the proposed control strategy with impulse effects ensures stable and reliable synchronization of the time-delayed NNs without stochastic perturbations.

**Fig. 3:**
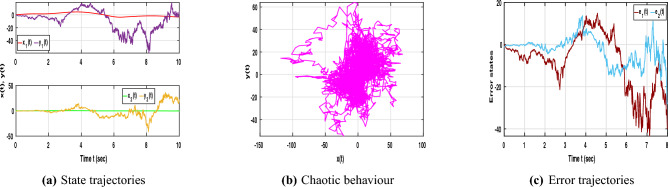
Dynamic behaviour of NNs (1) and (2) with stochastic influence and without control input.

With the inclusion of stochastic terms, Fig. 3a presents the state trajectories of NNs (1) and (2) under random disturbances without control input, where irregular and unsynchronized oscillations emerge due to stochastic perturbations. As shown in Fig. 3b, the chaotic trajectories display strong fluctuations and a noticeable loss of coherence, reflecting the system’s instability in the absence of control action. Furthermore, Fig. 3c demonstrates that the error trajectories vary widely instead of converging, confirming that stochastic effects hinder synchronization and emphasizing the importance of control intervention in maintaining stable dynamic behavior for time-delayed NNs.

**Fig. 4:**
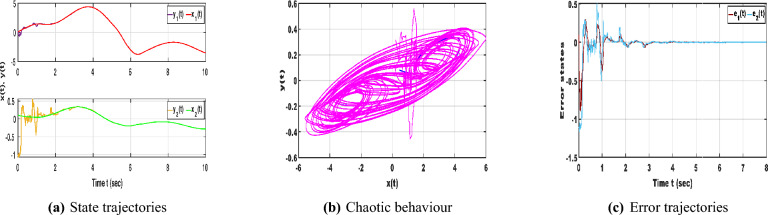
Dynamic behaviour of NNs (1) and (2) with stochastic effect and control input.

In Fig. 4a reveals that when both stochastic effects and control inputs are applied, the trajectories of the slave neural network (2) gradually align with those of the master system (1), confirming the effectiveness of the designed control law. In Fig. 4b, the phase portrait displays overlapping attractors, indicating that synchronization is achieved even under random disturbances. Additionally, Fig. 4c shows that the error states settle rapidly to zero despite the presence of noise, demonstrating the robustness and stability of the proposed control approach in managing stochastic perturbations within time-delayed NNs.

**Fig. 5:**
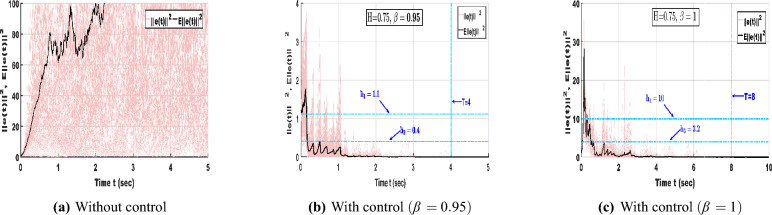
Error trajectories of NNs (8) without and with control under different orders $\beta = 0.95, 1$.

Assume that the parameters are chosen as $\hbar _1 = 1.1$, $\hbar _2 = 4$, $\hbar _3 = 0.4$, $\sigma = 0.2$, and $T = 4$. These parameter values satisfy the conditions specified in Definitions 1 and 2. Figure 5 illustrates the mean-square synchronization error trajectories of NNs (8) under different fractional orders with and without control input.

In Fig. 5a, when no control is applied, $E||e(t)||^2$ diverges rapidly and exhibits large stochastic oscillations, indicating that the system fails to maintain mean-square boundedness and cannot achieve synchronization under random perturbations. In contrast, Fig. 5b shows that when the proposed hybrid control is applied with the fractional order $\beta = 0.95$, the error trajectories decay sharply and remain confined within the admissible region determined by $(\hbar _1, \hbar _2, \hbar _3, \sigma , T) = (1.1, 4, 0.4, 0.2, 4)$. This satisfies both the MSFTSn and MSFTCSn criteria, confirming finite-time synchronization at approximately $T = 3\text {s}$. Furthermore, Fig. 5c depicts the case with integer order $\beta = 1$. When the order is fixed at $\beta = 1$, the error trajectories converge more slowly, reaching synchronization around $T = 8\text {s}$, and occupy a broader bounded region $(\hbar _1, \hbar _2, \hbar _3, \sigma , T) = (10, 30, 3.2, 0.5, 8)$. Therefore, compared with the integer-order system, the fractional-order case exhibits stronger damping, tighter boundedness, and faster convergence. This confirms that incorporating fractional dynamics enhances the system’s memory effect, accelerates error decay, and strengthens robustness against stochastic perturbations.

**Fig. 6:**
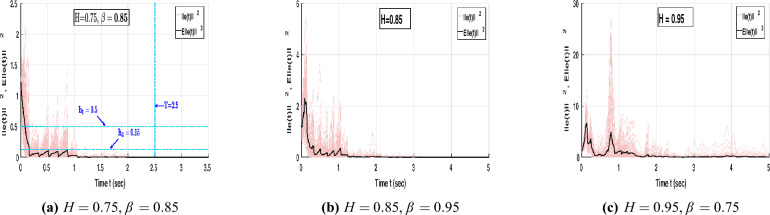
Dynamic behaviour of error NNs (8) under different Hurst parameters and fractional orders.

Under $H=0.75$, Fig. 6a shows that $E\Vert e(t)\Vert ^{2}$ remains within the region $(\hbar _{1},\hbar _{2},\hbar _{3},\sigma ,T)=(0.5,2.5,0.15,0.1,2.5)$ and achieves synchronization at $T=2.5\text {s}$ for fractional-order $\beta =0.85$. In comparison, Fig. 5b with $\beta =0.95$ is confined to (1.1, 4, 0.4, 0.2, 4) and synchronizes at $T=4\text {s}$. Hence, reducing the fractional order from $\beta =0.95$ to $\beta =0.85$ compresses the mean-square error bounds and shortens the synchronization time, indicating a stronger memory effect that accelerates convergence and improves resistance to stochastic fluctuations.

Furthermore, Fig. 6b and 6c illustrate the mean-square synchronization behavior of the error NNs (8) for different Hurst parameters $H=0.85$ and $H=0.95$ with $\beta =0.95$. Both cases confirm finite-time synchronization under the proposed control law; however, the synchronization region expands with increasing *H*. For $H=0.85$, smoother correlations in the fBm yield smaller fluctuation amplitudes and a tighter convergence region, whereas for $H=0.95$, stronger temporal persistence slightly enlarges the bounded region and introduces higher transient peaks. Overall, the results demonstrate that the designed controller effectively preserves boundedness and synchronization performance under varying stochastic dependencies.

$$\underline{{\textbf {Discontinuous case:}}}$$

**Fig. 7:**
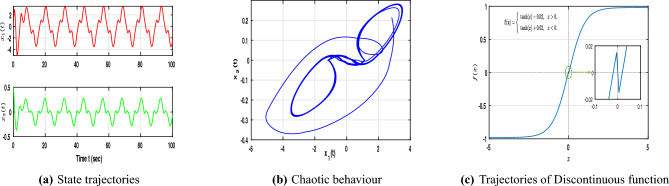
Dynamic behaviour of NNs (1) with discontinuous neuron activation function.

The matrices used in this case are identical to those used in the previously analyzed continuous case. Furthermore, set $\nu _1=0.67$, $\nu _2=-0.52$, $\nu _3=-0.42$, $\nu _4=0.21$, $\alpha =0.79$, and $\omega =1$. The discontinuous activation function employed in this case is defined as

$$\begin{aligned} f(x) ={\left\{ \begin{array}{ll} \tanh (x) - 0.02, & \text {if } x > 0, \\ \tanh (x) + 0.02, & \text {if } x < 0, \end{array}\right. } \end{aligned}$$

which adds a small shift on either side of the origin. This small offset creates a jump between the two parts of the function, making it discontinuous. As shown in Fig. 7, this discontinuity changes how the neuron responds, producing sharper transitions and more irregular, chaotic motion in the network states compared with the continuous case.

**Fig. 8:**
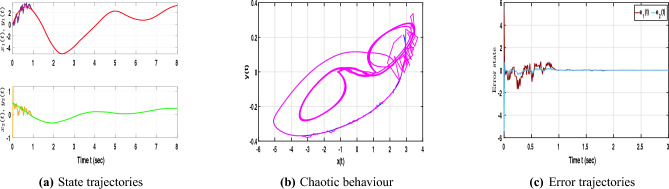
Dynamic behaviour of NNs (1) and (2) with discontinuous neuron activation function under hybrid control.

Using the above parameter settings and following the same LMI-based algorithm described earlier, the corresponding feasibility conditions are obtained through the MATLAB LMI Toolbox as follows:

$$\begin{aligned} \mathscr {P}=\begin{bmatrix} 0.0888 & -0.0701 \\ -0.0701 & 0.8777 \end{bmatrix}, \mathscr {Z}=\begin{bmatrix} 0.0048 & -0.0083 \\ -0.0083 & 0.1396 \end{bmatrix}, G=\begin{bmatrix} 7.6596 & 1.1181 \\ -0.2401 & 2.9173 \end{bmatrix}, \end{aligned}$$

and the control gain *K* is obtained as $$K=\begin{bmatrix} 91.8173 & 16.2353 \\ 7.0559 & 4.6200 \end{bmatrix}$$. Furthermore, Fig. 8 illustrates the state trajectories of NNs (1) and (2) with the discontinuous activation function under impulsive and control actions. It is evident that the trajectories of (2) closely follow those of (1), confirming successful synchronization and demonstrating the effectiveness of the proposed control strategy in handling discontinuous dynamics and stochastic behaviour.

**Fig. 9:**
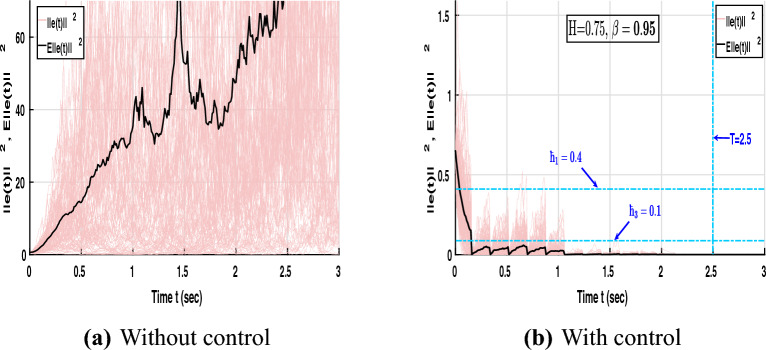
Dynamic behaviour of error NNs (8).

As shown in Fig. 9, the state trajectories of the error NNs (8) are illustrated both without and with control. In the uncontrolled case (9a), the trajectories $E\Vert e(t)\Vert ^{2}$ diverge due to stochastic excitation, exhibiting strong oscillations and loss of synchronization. In contrast, the controlled case (9b) demonstrates that the proposed aperiodic intermittent control achieves convergence within the finite terminal time $T = 2.5 \text {s}$. For the chosen parameters $\hbar _{1} = 0.4$, $\hbar _{2} = 1$, $\hbar _{3} = 0.1$, $\sigma = 0.05$, $H = 0.75$, and $\beta = 0.95$, the trajectories contract toward the origin, fulfilling the conditions of MSFTSn and MSFTCSn. This behavior confirms the robustness and finite-time synchronization capability of the proposed control scheme under stochastic perturbations.

Overall, the numerical simulations consistently validate the theoretical analysis established in Theorems 1 and 2, confirming that the proposed hybrid control approach guarantees both MSFTSn and MSFTCSn under stochastic disturbances. The continuous case described in Theorem 1 demonstrates that smooth control actions provide steady finite time convergence with well-damped system responses, ensuring stable synchronization. In contrast, the discontinuous case presented in Theorem 2 introduces switching dynamics that enhance robustness against stochastic perturbations and accelerate synchronization through faster error contraction. Furthermore, the comparison between the integer and fractional order systems shows that the fractional order structure possesses stronger memory and diffusion characteristics, resulting in faster convergence, reduced synchronization error, and a more compact bounded region. In addition, the control input plays a crucial role in stabilizing the network, as the trajectories diverge without control but rapidly converge when control is applied. Moreover, the fractional order $\beta$ and the Hurst parameter *H* significantly influence the system’s response. A smaller fractional order strengthens the memory and damping behavior, thereby accelerating the contraction of the mean square error, while a lower Hurst parameter produces smoother stochastic correlations and smaller fluctuation amplitudes. Therefore, the overall numerical evidence confirms that the proposed hybrid control, integrating both continuous and discontinuous mechanisms, achieves fast, stable, and noise-resilient synchronization in fractional-order stochastic neural networks.

### Remark 5

In earlier works^15–17^, synchronization of fractional-order delayed neural networks was typically achieved using single-action control strategies such as fractional feedback, impulsive, or matrix projection methods. Although these approaches ensured finite-time convergence, they often struggled to balance stability, convergence speed, and robustness under delays and uncertainties. To overcome these challenges, this study proposes a hybrid control framework that integrates continuous feedback with impulsive regulation. The continuous part maintains smooth error evolution and suppresses delay-induced oscillations, while the impulsive component provides rapid correction against stochastic disturbances. Leveraging the memory property of fractional derivatives, the scheme enhances damping and transient smoothness, achieving energy-efficient synchronization. Analytical results based on fractional Lyapunov functionals and impulsive differential inequalities confirm finite-time synchronization and mean-square stability, which are further supported by comparative simulations.

## Conclusion

Theoretical results established new sufficient conditions for MSFTSn and MSFTCSn in fractional-order stochastic delayed neural networks under a hybrid control framework. The analytical approach combined stochastic analysis with Lyapunov-based techniques, the fractional Gronwall inequality, and an improved Razumikhin method to derive finite-time synchronization criteria. The hybrid control, integrating continuous feedback with impulsive regulation, was crucial for compensating time delays, suppressing disturbances, and accelerating convergence, thereby enhancing synchronization efficiency with reduced control effort. Both continuous and discontinuous activation functions were effectively managed through smooth feedback and set-valued map theory. Numerical simulations confirmed the theoretical findings, demonstrating faster synchronization and stronger robustness, while the fractional-order formulation offered improved adaptability and realistic neural dynamics. Future research may extend this framework to coupled neural networks with symmetric saturation impulses to establish new synchronization criteria for large-scale interconnected systems.

## Data Availability

The authors declare that the data supporting the findings of this study are available within the paper. All simulation codes are available from the corresponding author on request.

## List of symbols

$\mathbb {R}, \mathbb {R}_{+}, \mathbb {Z}_{+}$: Sets of real numbers, positive real numbers, and positive integers
$\mathbb {R}^n$: *n*-dimensional real space equipped with the Euclidean norm $\Vert \cdot \Vert$
$\mathscr {A} > 0~(\mathscr {A} < 0)$: Matrix $\mathscr {A}$ is positive (negative) definite
$\mathscr {A}^{-1}, \mathscr {A}^{T}$: Inverse and transpose of matrix $\mathscr {A}$
$*$: Represents a symmetric block in a matrix
$\textrm{diag}$: Denotes a block diagonal matrix
$$\mathbb {E}(\cdot )$$: Expectation operator
$\{t_k\}_{k \in \mathbb {Z}_{+}}$: Increasing impulse sequence satisfying $\phi _1 \le t_{k+1}-t_k \le \phi _2$, $\forall k$
$\phi _1, \phi _2$: Minimum and maximum impulse dwell times on [0, *T*]
$B^{H}(t)$: Fractional Brownian motion with Hurst parameter $H \in (1/2,1)$
*C*(*H*, *W*): Set of continuous functions $\omega : H \rightarrow W$
*PC*(*H*, *W*): Set of piecewise continuous functions with finite discontinuities
$PC_{\tau } = PC([t_0-\tau ,t_0], \mathbb {R}^n)$: Space of piecewise continuous functions with delay $\tau$
$\Vert \psi \Vert _{\tau } = \sup _{s \in [t_0-\tau , t_0]} \Vert \psi (s)\Vert$: Supremum norm on $[t_0-\tau , t_0]$
$\mathfrak {K}$: Class of functions $b(\delta ) \in C(\mathbb {R}_{+}, \mathbb {R}_{+})$ satisfying $b(0)=0,~b(\delta )>0$ for $\delta >0$
$b(\delta )$: Strictly increasing function on $\mathbb {R}_{+}$
